# The correlation between raised body mass index and assisted reproductive treatment outcomes: a systematic review and meta-analysis of the evidence

**DOI:** 10.1186/s12978-018-0481-z

**Published:** 2018-02-27

**Authors:** Prasanna Raj Supramaniam, Monica Mittal, Enda McVeigh, Lee Nai Lim

**Affiliations:** 1 Oxford University Hospitals NHS Foundation Trust, John Radcliffe Hospital, Headley Way, Headington, Oxford, OX3 9DU UK; 2 Nuffield Department of Women’s and Reproductive Health, University of Oxford, Level 3, Women’s Centre, John Radcliffe Hospital, Oxford, OX3 9DU UK

**Keywords:** Body mass index (BMI), Assisted reproductive technology (ART), Overweight, Obese

## Abstract

**Background:**

Public funding for fertility services within the United Kingdom is limited, and therefore, strict guidance exists regarding who can be offered treatment under the National Health Service (NHS). Body mass index (BMI) is a universal criteria adopted by both the public and private sector.

This study addresses an important aspect of the impact of a raised BMI on fertility treatment outcomes. We standardise the analysis of the data by only including studies incorporating the WHO BMI criteria; the current reference point for clinicians and clinical commissioning groups in ascertaining which group of patients should receive treatment. This study is an update of the previous systematic review performed in 2010, with the inclusion of a larger number of cycles from central databases such as the Society for Assisted Reproductive Technology (SART).

**Methods:**

An electronic literature search was conducted through the Cochrane, Medline and Embase libraries. Data extraction for each outcome measure was pooled and expressed as an odds ratio with 95% confidence intervals. Where clinical heterogeneity was evident, the random effects model was used to calculate the risk ratio and a fixed effects model was used for the remaining studies. A *p* value < 0.05 was considered statistically significant.

**Results:**

A total of 49 studies have been identified and included in this systematic review. Overweight and obese (BMI ≥ 25 kg/m^2^) women have a statistically significant lower live birth rate (OR 0.81, 95% CI 0.74–0.89, *p* < 0.00001) following Assisted Reproductive Technology (ART) when comparisons are drawn to women with a normal BMI. An increase is also demonstrated in the number of miscarriages experienced by women with a BMI ≥ 30 kg/m^2^ (OR 1.52, 95% CI 1.28–1.81, *p* < 0.00001).

**Conclusion:**

Although this review concludes that a clear impact of BMI on ART outcomes is demonstrated, there remains questions as to the pathophysiology underlying these differences. This review supports the government’s stringent criteria regarding BMI categories under which NHS funding is made available for ART, through a clear description of poor reproductive outcomes in women with a BMI ≥ 30 kg/m^2^.

## Plain English summary

This study highlights the impact of an overweight or obese female partner on fertility treatment outcomes, in particular focusing on IVF. Women who are overweight or obese have been shown to be less likely to have a life birth outcome from an IVF cycle. They are also more likely to suffer from early miscarriages whilst undergoing fertility treatments.

## Main manuscript

The correlation between raised body mass index and assisted reproductive treatment outcomes: A systematic review and meta-analysis of the evidence.

## Background

Obesity is a major challenge for today’s clinicians. In 2016, the World Health Organisation (WHO) [1] stated that a staggering 39% of adults aged > 18 years fell into the overweight category, of which 40% were accounted for by women. Furthermore, 13% of the adult population were documented to be obese, with women accounting for 15% (WHO Global Health Observatory Data 2016). A raised body mass index (BMI) has been linked to a number of medical comorbidities, as well as being implicated in having a detrimental impact on the reproductive capacity of women in particular. Women who fall into high BMI categories can present with hypothalamic-pituitary ovarian dysfunction and thus, low fecundity rates. In 2011, Rittenberg et al., [2] concluded that women with a BMI ≥ 25.0 kg/m^2^ had a lower live birth rate through assisted reproductive treatments (ART) compared with women of a normal BMI. This has been further supported by multiple large studies evaluating the impact of BMI on ART outcomes.

The WHO classification of BMI is widely referred to, and provides standardisation for comparison of research outcomes. A documented BMI of 18.5–24.9 kg/m^2^ is considered normal and healthy and the preferred range. A BMI of 25–29.9 kg/m^2^ refers to overweight and a BMI ≥ 30 kg/m^2^ is considered obese. The latter range is further subdivided into Class 1 (30.0–34.9 kg/m^2^), Class 2 (35.0–39.9 kg/m^2^) and Class 3 (≥ 40.0 kg/m^2^).

This paper, considers the current evidence regarding the impact of raised BMI on outcomes following ART treatment. A systematic review and meta-analysis of the available evidence will help provide or refute the current recommendations from the government regarding the allocation of resources for fertility treatment.

## Methods

### Search strategy

Literature searches were conducted through the Cochrane, Embase and Medline libraries (1966–2017). The medical subject headings (MeSH) were generated for two categories: 1. Body mass index (BMI, overweight, obesity); 2. in vitro fertilisation (IVF)/ intracytoplasmic sperm injection (ICSI) (embryo, embryo transfer, ART). All identified papers were reviewed by two authors (PRS and MM) independently. All discrepancies, regarding inclusion or exclusion of the data were discussed with a final decision mutually agreed upon.

### Study inclusion and exclusion criteria

All relevant published studies reporting on the effects of BMI on IVF and ICSI pregnancy outcomes were included. Studies that reported donor cycles, conception by natural cycles, intrauterine insemination, waist hip ratio, and non-WHO classification of BMI were excluded. In addition, studies reporting on the effects of paternal body mass index on IVF/ ICSI outcomes were also excluded.

### Outcome measures

The primary outcome measure assessed was live birth rate following an IVF/ ICSI cycle. Secondary outcome measures included: clinical pregnancy rate; and, miscarriage rate. The presence of a gestational sac on an ultrasound scan at least four weeks following on from an embryo transfer was used as confirmation for a clinical pregnancy. The clinical pregnancy rate was calculated per IVF/ ICSI cycle. For the purpose of this review, miscarriage was defined as pregnancy loss ≤ 20 weeks gestation. The miscarriage rate was calculated per clinical pregnancy.

### Statistical analysis

Data extraction for each outcome measure was pooled and expressed as an odds ratio (OR) with a 95% confidence interval (CI). Clinical heterogeneity (I^2^) [3] was considered significant when the I^2^ value was < 50%. Where clinical heterogeneity was evident, the random effects model (DerSimonian and Laird, 1986) was used to calculate the risk ratio, and clinical heterogeneity was explored by comparing the variation in studies, such as, study design, study quality and interventions. Particular care was taken to further evaluate studies with similar first authors to avoid heterogeneity in the study population. For the remaining pooled data, the fixed effect model [4] was used to calculate the risk ratio. Statistical analysis was performed using the RevMan 5.3 software. A *p* value < 0.05 was considered statistically significant.

## Results

**Fig. 1:**
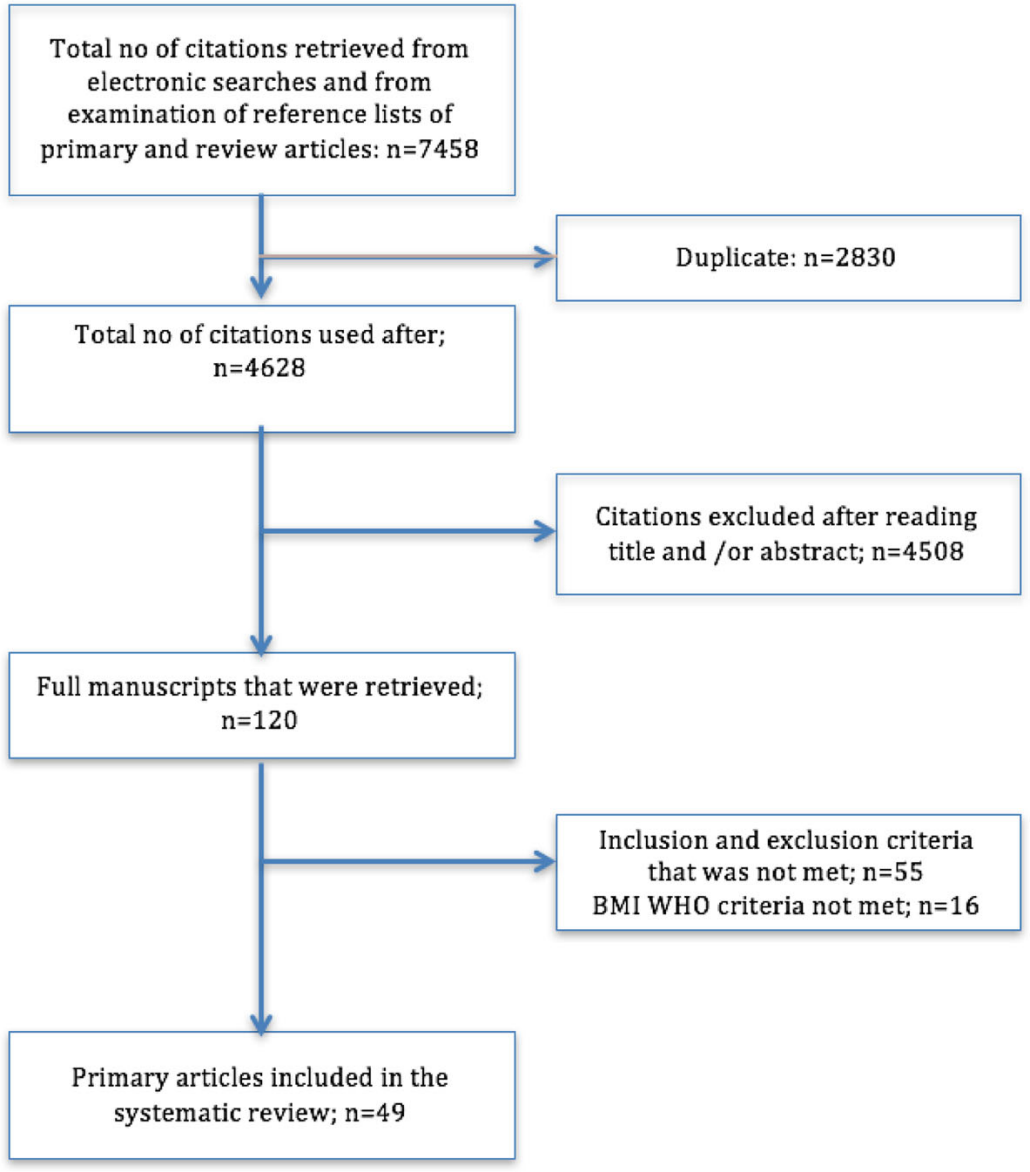
Flow chart for literature search and study selection

The search strategy yielded 7458 electronic citations (Fig. 1). Of this, 2830 were removed secondary to duplications. Titles and abstracts were reviewed for the remaining 4628 publications. After screening of the titles and abstracts, 4508 publications were further excluded. Full manuscripts were obtained for the remaining 120 articles. A further 16 articles were excluded as they did not use the WHO classification for BMI categories. A further 55 articles were excluded as per the inclusion exclusion criteria. The remaining 49 articles met all requirements and were included in this systematic review and meta-analysis (Table 1).

**Table 1:** Details of included studies

Study	Methodology (population size)	Intervention	Exclusion Criteria	BMI Categories (kg/m^2^) and numbers	Outcome Measures
Fedorcsak et al. 2000 [8] (1996–1998)	Retrospective Cohort study (383 women)	IVF/ICSI	12 patients excluded as incomplete data	< 25.0 (304 women) ≥25.0 (79 women)	Live birth rate Fertilization rate No of oocytes retrieved Abortion rate
Wittemer et al. 2000 [9] (1997–1998)	Retrospective study (398 women)	IVF/ICSI	None stated	< 20.0 (87 women) 20.0–25.0 (222 women) ≥ 25.0 (89 women)	Pregnancy rate Delivery rate Miscarriage rate
Wang et al. 2000 [10] (1987–1998)	Retrospective study (3586 women)	IVF/ICSI and GIFT	None stated	< 20.0 (441 women) 20.0–24.9 (1910 women) 25.0–29.9 (814 women) 30.0–34.9 (304 women) ≥35.0 (117 women)	Probability of achieving at least one pregnancy
Loveland et al. 2001 [11] (1997–1999)	Retrospective study (139 women / 180 cycles)	IVF	Women > 40 years of age, blastocyst or frozen embryo transfer, donor cycles	≤25 (70 women / 87 cycles) > 25 (69 women / 93 cycles)	Number of oocytes Clinical pregnancy rate Spontaneous abortion Ongoing pregnancy rate
Wang et al. 2001 [12] (1987–1999)	Cohort study (1018 women)	IVF/ICSI/GIFT	Women whose BMI or PCOS status was not assessed	< 20.0 (112 women) 20.0–24.9 (509 women) 25.0–29.9 (231 women) 30.0–34.9 (116 women) ≥35.0 (50 women)	Spontaneous abortion
Wang et al. 2002 [13] (1987–1999)	Retrospective analysis (2349 women)	IVF/ICSI/GIFT	Ectopic pregnancy, late pregnancy, women whose BMI was measured >/= 1 year before pregnancy	< 18.5 (70 women) 18.5–24.9 (1508 women) 25–29.9 (503 women) 30–34.9 (198 women) ≥35 (70 women)	Spontaneous miscarriage
Winter et al. 2002 [14] (1994–1999)	Cohort (1123 women / 1196 cycles)	IVF/ICSI/GIFT		< 18.5 (26 women) 18.5–25.0 (701 women) 25.1–30.0 (243 women) 30.1–35.0 (107 women) > 35.0 (46 women)	Early pregnancy loss
Doody et al. 2003 [15] (2000–2003)	Retrospective analysis (822 retrievals)	IVF/ICSI	Donor cycles, age > 40 years	< 25 (460 women) 25–29.9 (194 women) 30–34.9 (89 women) > 35 (79 women)	Pregnancy rate Implantation rate No of oocytes No of embryos transferred Ongoing pregnancy rate
Fedorscak et al. 2004 [16] (1996–2002)	Retrospective Study (2660 women / 5019 cycles)	IVF/ICSI	None stated	< 18.5 (76 women/136 cycles) 18.5–24.9 (1839 women/3457 cycles) 25.0–29.9 (504 women/963 cycles) ≥30.0 (241 women/463 cycles)	No of oocytes collected No of embryo transferred No of embryo transfers No of biochemical pregnancies Early pregnancy loss Miscarriage (6–12 weeks), (> 12 weeks) Ectopic pregnancy Stillbirth Live birth rate Dose of FSH Duration of FSH
Ryley et al. 2004 [17]	Retrospective study (6827 cycles)	IVF	Women with BMI > 40	< 20.0 (466 cycles) 20.0–24.9 (3605 cycles) 25.0–29.9 (1632 cycles) 30.0–34.9 (724 cycles) =35 (400 cycles)	Clinical pregnancy rate No of oocytes
Van Swieten et al. 2005 [18]	Observational (162 women/ 288 cycle)	IVF/ICSI	None stated	< 25 (101 women) 25–30 (32 women) > 30 (29 women)	Fertilisation rate No oocytes retrieved Clinical pregnancy rate Abortion rate
Hammadeh et al. 2005 [19]	Prospective (52 women)	IVF	None stated	≤25.0 (28 women) > 25.0 (24 women)	Pregnancy rate
Dechaud et al. 2006 [20]	Prospective study (573 women/ 789 cycles)	IVF/ICSI	Women with a history of uterine surgery, hydrosalpinges evidenced by ultrasonography, three or more failed attempts at IVF, frozen-thawed cycles, women undergoing pre-implantation diagnosis and those using a protocol other than the long protocol	< 20 (186 women/ 264 cycles) 20–25 (283 women/ 394 cycles) 25–30 (68 women/ 83 cycles) ≥30 (36 women/ 48 cycles)	Duration of ovarian stimulation Dose of FSH Implantation rate No of oocytes Fertilization rate Clinical pregnancy rate Miscarriage rate
Dokras et al. 2006 [21] (1995–2005)	Retrospective Study (1293 women)	IVF/IVF with ICSI	Women > 38 years of age, day 2 transfer cycles, cryopreserved embryo transfers, donor oocyte cycle, gamete intrafallopian transfer and zygote intrafallopian transfer cycles	< 25 (683 women) 25–29.9 (295 women) 30.0–39.9 (236 women) ≥40 (79 women)	No of follicles aspirated Fertilization rate No of embryo(s) transferred Clinical pregnancy rate Miscarriage rate Delivery rate Days of stimulation
Mitwally et al. 2006 [22]	Cohort (183 cycles)	IVF	None stated	< 25.0 (102 cycles) ≥25.0 (81 cycles)	Clinical pregnancy rate
Metwally 2007 [23] (2001–2006)	Retrospective analysis (426 women)	IVF/ICSI	Cycles on women whose BMI was unrecorded	19–24.9 (241 women) 25–29.9 (113 women) ≥30 (72 women)	Fertilization rate Clinical pregnancy rate Dose of FSH Duration of FSH No of oocytes collected
Esinler et al. 2008 [24]	Retrospective Study (775 women/ 1113 cycles)	ICSI	Freeze-thaw cycles, female age > 40, presence of PCOS, history of irregular menstrual cycle and suspected poor ovarian response	18.5–24.9 (451 women/ 627 cycles) 25.0–29.9 (222 women/ 339 cycles) ≥30.0 (102 women/ 147 cycles)	Clinical pregnancy rate Fertilization rate No of miscarriages No of oocytes Dose of FSH Duration of FSH
Martinuzzi et al. 2008 [25] (2004–2006)	Retrospective study (417 women)	IVF	Women > 36 years of age, cycle day-3	< 18.5 (21 women) 18.5–24.9 (267 women) 25.0–29.9 (77 women) ≥30 (52 women)	No of oocytes Fertilization rate Implantation rate Clinical pregnancy rate Ongoing pregnancy rate
Moini et al. 2008 [26] (2002–2003)	Cross-sectional study (287 women)	IVF/ICSI	Women who did not have polycystic ovary syndrome, age > 40 years, BMI < 20, women with hypo/hyperthyroidism, hyperprolactinemia and diabetes type 1	20–25 (133 women) 25.1–30 (117 women) > 30 (37 women)	No of oocytes No of transferred embryos Clinical pregnancy rate Miscarriage rate
Sneed et al. 2008 [27] (2005–2006)	Retrospective analysis (1273 women)	IVF	Frozen cycles, donor oocyte or gestational surrogacy cycles, age > 44 years	< 18.5 (28 women) > 18.5–24.9 (613 women) > 25–29.9 (325 women) > 30 (307 women)	No of oocytes No of embryo transfers Fertilization rate Implantation rate Spontaneous abortion Clinical pregnancies Live birth rate
Ozgun et al. 2009 [28] (2006–2007)	Prospective study (604 women)	ICSI	Women > 42 years old, medical co-morbidities such as diabetes mellitus, hyper or hypothyroidism, basal FSH > 15 IU/L, thawed embryo transfer cycles, history of prior ovarian surgery, poor responders, couples with more than one etiology for their infertility	< 18.5 (10 women) 18.5–24.9 (232 women) 25–29.9 (229 women) 30–35.9 (111 women) ≥36 (22 women)	No of Pregnancy Total FSH dosage
Sathya et al. 2010 [29]	Retrospective study (308 women)	IVF	Women > 40 years of age, FSH > 10 mIU/ml	< 25 (88 women) 25–30 (147 women) > 30 (73 women)	No of embryos transferred Clinical pregnancy rate Missed abortion rate Multiple pregnancy rate Ectopic pregnancy rate Implantation rate Gonadotrophin dosage
Zhang et al. 2010 [30] (2002–2008)	Retrospective study (2628 women)	IVF/ICSI	Patients with severe endometriosis (lll and IV stage) diagnosed by laparoscopy, more than two failed previous attempts, preimplantation diagnosis cycles, frozen thawed cycles, protocols other than the long protocol	18.5–24.9 (2222 women) 25.0–29.9 (379 women) ≥30.0 (27 women)	No of oocytes Fertilization rate Pregnancy rate Early pregnancy loss rate Ectopic pregnancy Miscarriage rate Live birth rate Days of FSH stimulation Dosage of FSH stimulation Ongoing pregnancy rate
Bellver et al. 2010 [31] (2001–2007)	Retrospective study (6500 cycles)	IVF/ICSI	None stated	< 20 (669 women / 1070 cycles) 20–24.9 (2620 women/ 3930 cycles) 25–29.9 (676 women/ 1081 cycles) ≥ 30 (262 cycles/ 419 cycles)	Total dose of gonadotrophin No of oocytes Fertilization rate No of embryos transferred Implantation rate Pregnancy rate Clinical pregnancy rate Clinical and global miscarriage rate Live birth rate
Vilarino et al. 2010 [32] (2008)	Retrospective (208 cycles/ 191 women)	IVF/ICSI	Frozen and donor oocyte-derived cycles	< 25 (137 cycles) ≥25 (71 cycles)	Fertilisation rate No of transferred embryos Pregnancy rate Early pregnancy loss Clinical miscarriage rate Ectopic pregnancy Live birth rate Dosage of FSH
Farhi et al. 2010 [33] (2006–2007)	Retrospective study (233 women/ 233 cycles)	IVF	Women ≥38 years of age, other than 2 high-quality embryos, ≥3 previous IVF attempts, women with hydrosalpinx, fibroid uterus, congenital uterine anomaly and chronic illness	≤25.0 (160 women) > 25.0 (73 women)	Live birth rate Pregnancy rate No of oocytes Fertilization rate
Davies et al. 2010 [34] (2008–2009)	232 cycles	IVF	Donor egg, gestational carrier and pre-implantation genetic diagnosis cycles	< 25.0 (176 cycles) > 25.0 (56 cycles)	Fetal heartbeat rates
Funabiki et al. 2011 [35] (2006–2010)	Retrospective study (859 women)	IVF	None stated	< 18.5 (152 women) 18.5–25.0 (648 women) ≥25.0 (59 women)	Pregnancy rate Ongoing pregnancy rate Miscarriage rate No of oocytes
Hill et al. 2011 [36]	Prospective study (117 women)	IVF	Women > 42 years of age, patients with elevated FSH levels (≥12 mIU/mL)	< 25.0 (58 women) ≥25.0 (59 women) < 30.0 (96 women) ≥30.0 (21 women)	Live birth rate Pregnancy rate Implantation rate No of oocytes No of embryo transferred Days of stimulation
Pinborg et al. 2011 [37] (2005–2006)	Cohort study (487 women/ 1417 cycles)	IVF/ ICSI/ FET	Patients undergoing intrauterine insemination cycles, patients with an existing child from fertility treatment, couples who had adopted a child in the 12th month follow-up period and couples who had no treatment during the first 12 months of follow up	< 18.5 (20 women) 18.5–24.9 (305 women) 25.0–29.9 (103 women) ≥30.0 (59 women)	Fertilization rate No of oocytes Biochemical pregnancy rate Ectopic pregnancy rate Ongoing pregnancy rate Miscarriage rate Live birth rate Dose of gonadotrophin stimulation
Parker et al. 2011 [38] (2010–2011)	Retrospective study (995 patients)	IVF/ICSI	None stated	< 18.5 (18 women) 18.5–24.9 (475 women) 25–29.9 (241 women) > 30 (221 women)	No of oocytes Clinical pregnancy rate Implantation rate Ongoing pregnancy rate Total FSH dosage No of embryo transferred
Rittenberg et al. 2011 [39] (2006–2010)	Cohort Study (413 women)	IVF/ICSI	Women > 40 years, BMI < 18.5, BMI > 35, pre-implantation genetic diagnosis, donor oocyte or embryos frozen for fertility preservation prior to cancer therapy cycles, mullerian duct anomalies, monozygotic twin gestations	18.5–24.9 (192 women) ≥25 (133 women)	Oocyte fertilisation rate No of oocytes Clinical pregnancy rate Live birth rate Miscarriage rate Duration of stimulation
Singh et al. 2011 [40] (2008–2010)	Retrospective Study (328 women/ 342 cycles)	IVF/ICSI	Women with confounding factors for poor response, endometrial pathologies, hydrosalpinx, ≥3 previous failed attempts, frozen thawed cycles	< 18.5 (26 women) 18.5–24.9 (141 women) 25–29.9 (131 women) > 30 (18 women)	Fertilisation rate Pregnancy rate Total dose of FSH Total days of stimulation No of oocytes retrieved Fertilization rate Clinical pregnancy rate
Luke et al. 2011 [41] (2007–2008)	Historical cohort study (152,500 cycles)	IVF	Women whose height and weight were not recorded, gestational carrier cycles, research or embryo banking with no outcome reported	< 18.5 (4254 cycles) 18.5–24.9 (86,860 cycles) 25–29.9 (35,452 cycles) 30.0–34.9 (15,406 cycles) 35.0–39.9 (6920 cycles) 40.0–44.9 (2513 cycles) 45.0–49.9 (805 cycles)	Pregnancy rate Fetal death or stillborn
Chavarro et al. 2012 [42] (2004–2011)	Prospective study (170 women/ 233 cycles)	IVF/ICSI	Women < 18 and > 45 years of age	< 20 (22 women) 20–22.4 (47 women) 22.5–24.9 (42 women) 25–29.9 (35 women) ≥30 (24 women)	Clinical pregnancy rate Total gonadotrophin dose Fertilization rate Clinical pregnancy rate Live birth rate
Galal et al. 2012 [43]	Prospective cohort (220 women)	ICSI	None stated	< 25.0 (110 women) > 25.0 (110 women)	No of oocytes Fertilization rate Clinical pregnancy rate No of embryos transferred
Werner et al. 2012 [44] (2008–2012)	Retrospective study (355 women)	IVF	None stated	< 18.5 (13 women) 18.5–24.9 (209 women) 25.0–29.9 (88 women) > 30.0 (45 women)	Pregnancy rate Clinical implantation rate Sustained implantation rate
Zander-Fox et al. 2012 [45] (2006–2007)	Retrospective study (2089 cycles)	IVF/ICSI	Women > 38 years of age, natural and donor cycles	18.5–24.9 (1065 cycles) 25.0–29.9 (486 cycles) 30.0–34.9 (244 cycles) 35.0–39.9 (144 cycles) ≥40.0 (118 cycles)	No of oocytes Fertilisation rate Live delivery Clinical pregnancy No of oocytes
Ozgun et al. 2012 [46] (2005–2010)	Retrospective cohort (935 women)	ICSI	No exclusion criteria	< 18.5 (18 women) 18.5–24.9 (398 women) 25–29.9 (355 women) ≥30 (164 women)	Clinical pregnancy rateNo of oocytes Miscarriage rate Total gonadotrophin dose
Ramezanzadeh et al. 2012 [47] (2010–2011)	Prospective study (236 women)	IVF	Male factor infertility according to the WHO criteria, presence of systemic disease, age < 18 years or > 40 years and donor oocytes	< 25 (93 women) 25–30 (94 women) > 30 (49 women)	No of oocytes Fertilization rate No of embryo transferred Biochemical pregnancies Clinical pregnancy rate Implantation rate
Moragianni et al. 2012 [48] (2007–2008)	Retrospective cohort study (4609 women)	IVF/ IVF-ICSI	Women < 20 years and > 47 years of age, donor oocytes, gestational surrogacy, cryopreserved embryos or those that lacked BMI documentation	< 18.5 (92 women) 18.5–24.99 (2605 women) 25.0–29.99 (1027 women) 30.00–34.99 (477 women) 35.00–39.99 (275 women) > 40.0 (133 women)	No of oocytes retrieved Duration of stimulation Total dosage of gonadotrophin No of embryo transferred Implantation Clinical pregnancy Biochemical pregnancy Global miscarriage Ectopic pregnancy Live birth Multiple birth
Bailey et al. 2014 [49] (2001–2010)	Retrospective Cohort Study (79 women / 101 cycles)	IVF/ICSI	Women < 40 years of age, height and weight measurements > 3 months from the start of cycle, in-vitro maturation, FSH > 10 mIU/mL, uncontrolled thyroid disease, history of chemotherapy or radiation exposure, recurrent pregnancy loss, uterine factor, balanced translocation in either partner, surgically documented endometriosis or pelvic adhesions, history of pelvic inflammatory disease, adenomyosis and submucosal myoma	18.7–24.9 (51 cycles) 25.0–29.9 (19 cycles) ≥30.0 (31 cycles)	Chemical pregnancy Miscarriage Clinical Pregnancy Live Birth rate Duration of stimulation of gonadotrophin Dosage of gonadotrophin No of oocytes retrieved
Schliep et al. 2014 [50] (2005–2010)	Prospective Cohort Study (721 women)	IVF/ICSI	Men with non-obstructive azoospermia	< 18.5 (32 women) 18.5–24.9 (407 women) 25–29.9 (147 women) 30–34.9 (72 women) ≥35 (63 women)	Fertilization rate Pregnancy rate Live birth rate
Cai et al. 2017 [51] (2013–2014)	Retrospective Cohort Study (4401 women / 4798 fresh transfer cycles	IVF/ICSI	Mild stimulation cycles, natural cycles and luteal-phase stimulation cycle, patients with diabetes, glucose intolerance and thyroid abnormality	< 18.5 (886 cycles) 18.5–24.9 (3642 cycles) ≥25 (670 cycles)	Fertilization rate Live birth rate Miscarriage rate Dosage of gonadotrophin
Ozekinci et al. 2015 [52] (2008–2013)	Retrospective Cohort Study (298 women)	IVF-ICSI	Underweight women, women > 38 years of age, transfer of > 2 embryos, frozen cycles	18.5–24.9 (164 cycles) 25–29.9 (70 cycles) ≥30 (64 cycles)	Dosage of gonadotrophin Duration of stimulation
Caillon et al. 2015 [53] (2006–2009)	Retrospective study (582 women)	IVF-ICSI	Underweight women	18.5–24.9 (409 women) ≥25 (149 women)	Dosage of gonadotrophin Implantation rate Miscarriage rate Live birth rate
Provost et al. 2016 [54] 2008–2010	Retrospective Cohort Study (239,127 cycles)	IVF	Women with a height < 48 in. and weight < 70 pounds	< 18.5 (7149 cycles) 18.5–24.9 (134,588 cycles) 25–29.9 (54,822 cycles) 30–34.9 (24,922 cycles) 35–39.9 (11,747 cycles) 40–44.9 (4084 cycles) 45–49.9 (1292 cycles) > 50 (463 cycles)	Implantation rate Clinical pregnancy rate Miscarriage rate Live birth rate
Russo et al. 2017 [55] 2010–2014	Retrospective Cohort Study (520 women)	Not specified	Congenital uterine anomalies, endometrial polyps, intrauterine synechiae, adenomyosis, intra-cavity fibroids, hydrosalpinges, donor cycles, poor quality embryos, cleavage stage embryos, and women > 40 years	< 20 (51 women) 20–24.9 (294 women) 25–29.9 (64 women) 30–39.9 (58 women) ≥40 (54 women)	Miscarriage rate Clinical pregnancy rate Live birth rate Dosage of gonadotrophin
Christensen et al. 2016 [56] (1999–2009)	Retrospective Cohort Study (5342 cycles)	IVF/ICSI	Missing information on BMI or treatment type, premature ovulation before oocyte retrieval, intrauterine insemination cycles	< 18.5 (158 cycles) 18.5–24.9 (3539 cycles) 25–29.9 (1171 cycles) ≥30 (474 cycles)	Dosage of gonadotrophin Clinical pregnancy rate

*BMI* Body Mass Index, *IVF* in vitro fertilization, *ICSI* intracytoplasmic sperm injection, *OHSS* ovarian hyperstimulation syndrome, *GIFT* gamete intra-Fallopian transfer, *HCG* human chorionic gonadotrophin, *FSH* follicle stimulation hormone

### Primary outcome measure

#### Life birth rate per IVF/ ICSI cycle

**Fig. 2:**
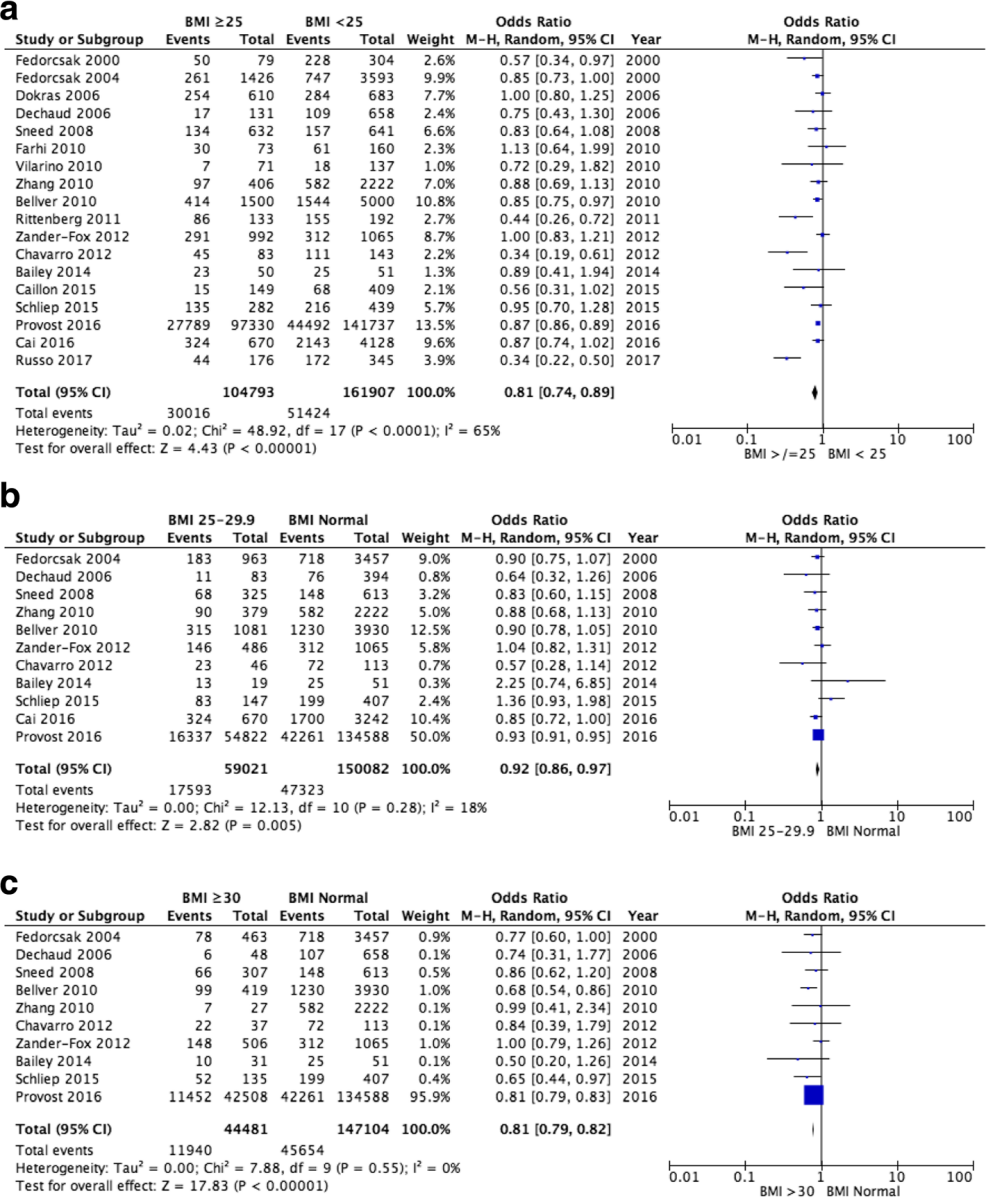
Meta-analysis of live-birth rate: (**a**) BMI ≥25 kg/m^2^ versus BMI < 25 kg/m^2^; (**b**) Normal BMI versus BMI 25–29.9 kg/m^2^; (**c**) Normal BMI versus BMI ≥30 kg/m^2^

In women with a BMI ≥25 kg/m^2^ versus BMI < 25 kg/m^2^, a total of 14 studies were pooled and a statistically significant reduction in the live birth rate (OR 0.81, 95% CI 0.74–0.89, *p* < 0.00001; Fig. 2a) was seen. There was significant heterogeneity between the included studies (I^2^ = 65%).

A total of 11 studies compared women with a normal BMI against those who were overweight (BMI 25–29.9 kg/m^2^). An analysis of the pooled data showed a statistically significant reduction in the live birth rate in women with a BMI 25–29.9 (OR 0.92, 95% CI 0.86–0.97, *p* = 0.005; Fig. 2b). No significant heterogeneity (I^2^ = 18%) was documented.

Data for women with a normal BMI versus BMI ≥ 30 kg/m^2^ came from the pooling of 10 studies. The live birth rate for women with a BMI ≥30 kg/m^2^ was statistically significantly lower than for women with a normal BMI (OR 0.81, 95% CI 0.79–0.82, *p* < 0.00001; Fig. 2c). No significant heterogeneity (I^2^ = 0%) was detected in the data source.

### Secondary outcome measures

#### Clinical pregnancy rate

**Fig. 3:**
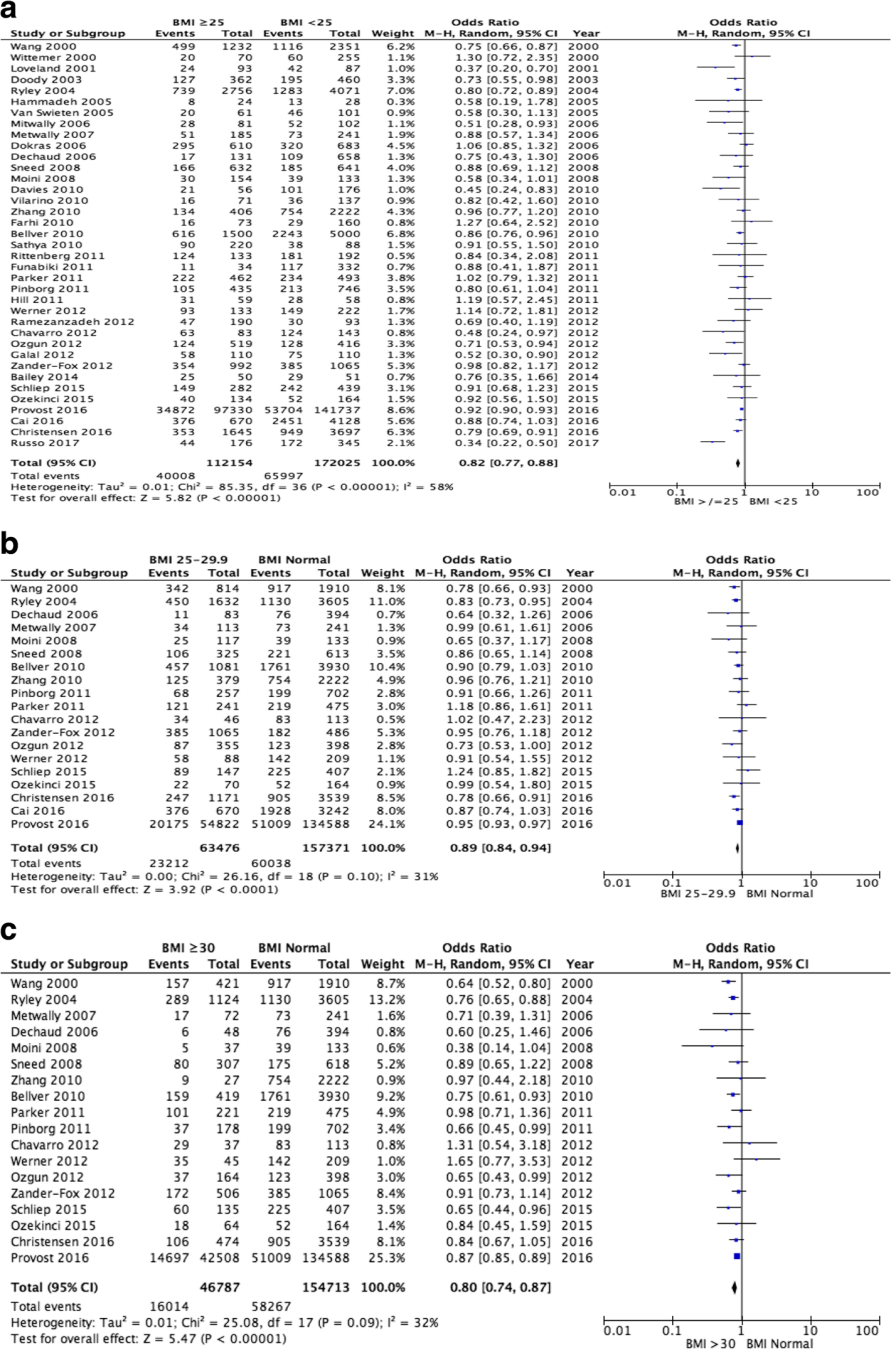
Meta-analysis of clinical pregnancy rate: (**a**) BMI ≥25 kg/m^2^ versus BMI < 25 kg/m^2^; (**b**) Normal BMI versus BMI 25–29.9 kg/m^2^; (**c**) Normal BMI versus BMI ≥30 kg/m^2^

A total of 37 studies were pooled for BMI < 25 kg/m^2^ versus BMI ≥25 kg/m^2^. A statistically significant reduction in the clinical pregnancy rate was demonstrated for women with a BMI ≥25 kg/m^2^ (OR 0.82, 95% CI 0.77–0.88, *p* < 0.00001; Fig. 3a). However, there was significant heterogeneity (I^2^ = 58%, p < 0.00001) between the studies analysed.

A statistically significant reduction in the clinical pregnancy rate was demonstrated for women with a BMI between 25 and 29.9 kg/m^2^ when compared to women with a normal BMI (19 studies pooled, OR 0.89, 95% CI 0.84–0.94, *p* < 0.00001; Fig. 3b). No significant heterogeneity (I^2^ = 31%) was seen between the studies.

Pooled analysis from 18 studies demonstrated a statistically significant reduction in the clinical pregnancy rate for women with a BMI ≥30 kg/m^2^ when compared to women with a normal BMI (OR 0.80, 95% CI 0.74–0.87, *p* < 0.00001; Fig. 3c). There was no significant heterogeneity (I^2^ = 32%) present between the studies.

#### Miscarriage rate

**Fig. 4:**
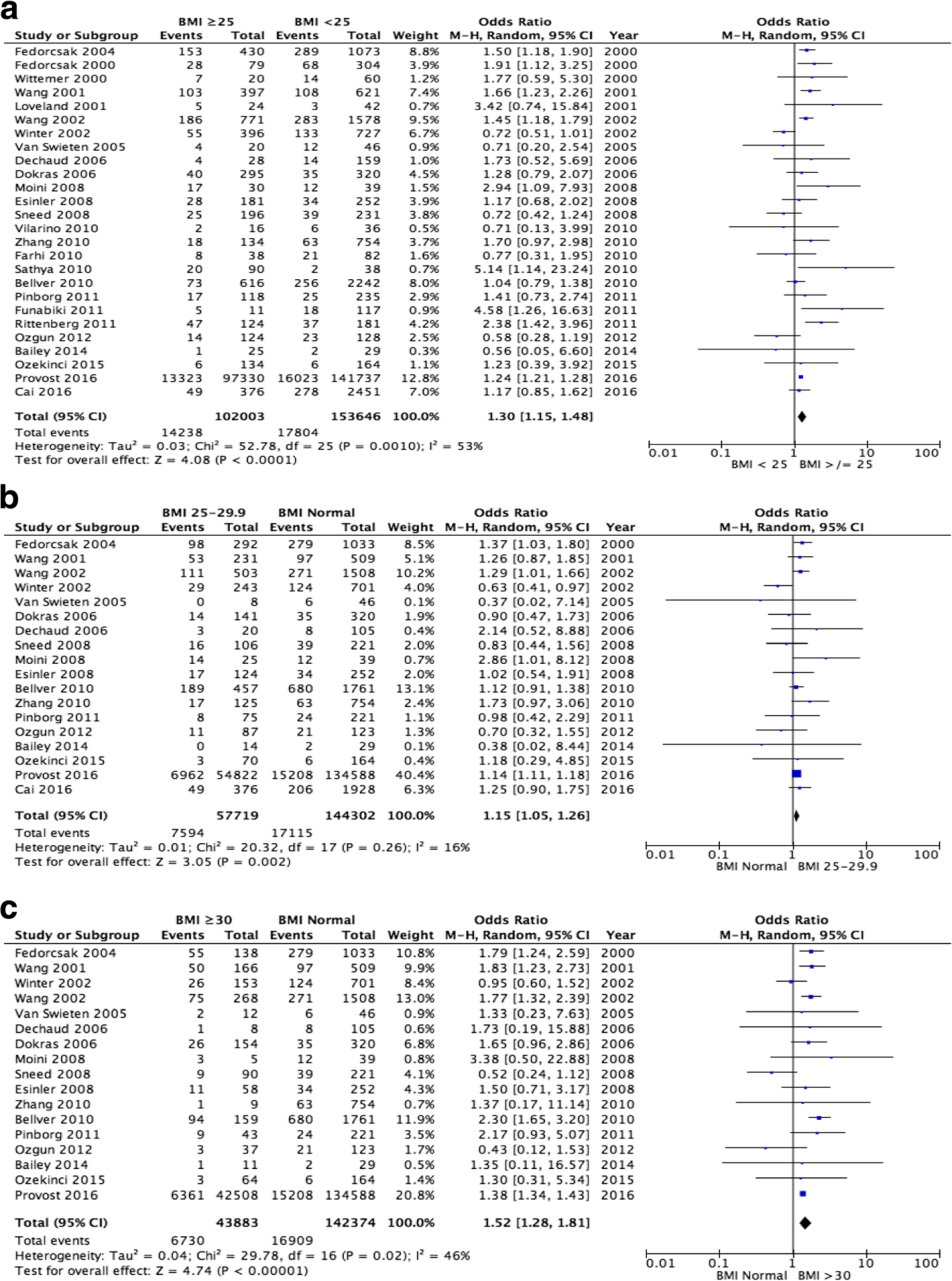
Meta-analysis of miscarriage rate: (**a**) BMI ≥25 kg/m^2^ versus BMI < 25 kg/m^2^; (**b**) Normal BMI versus BMI 25–29.9 kg/m^2^; (**c**) Normal BMI versus BMI ≥30 kg/m^2^

An increased risk of miscarriage is demonstrated in women with a BMI ≥25 kg/m^2^ when compared to women with a BMI < 25 kg/m^2^ (26 studies pooled, OR 1.30, 95% CI 1.15–1.48, *p* < 0.0001; Fig. 4a). However, significant heterogeneity (I^2^ = 53%, *p* = 0.0001) was seen between the studies.

Women with a BMI 25–29.9 kg/m^2^ were also more likely to have a miscarriage when compared to women with a normal BMI (18 studies pooled, OR 1.15 95% CI 1.05–1.26, *p* = 0.002; Fig. 4b). There was no significant clinical heterogeneity (I^2^ = 16%) in this group.

The risk of miscarriage is further increased in women with a BMI ≥30 kg/m^2^ when compared to women who fall into a normal BMI category (17 studies pooled, OR 1.52, 95% CI 1.28–1.81, *p* < 0.00001; Fig. 4c). No significant heterogeneity (I^2^ = 46%) was demonstrated between the studies.

#### Dosage of gonadotrophin stimulation

**Fig. 5:**
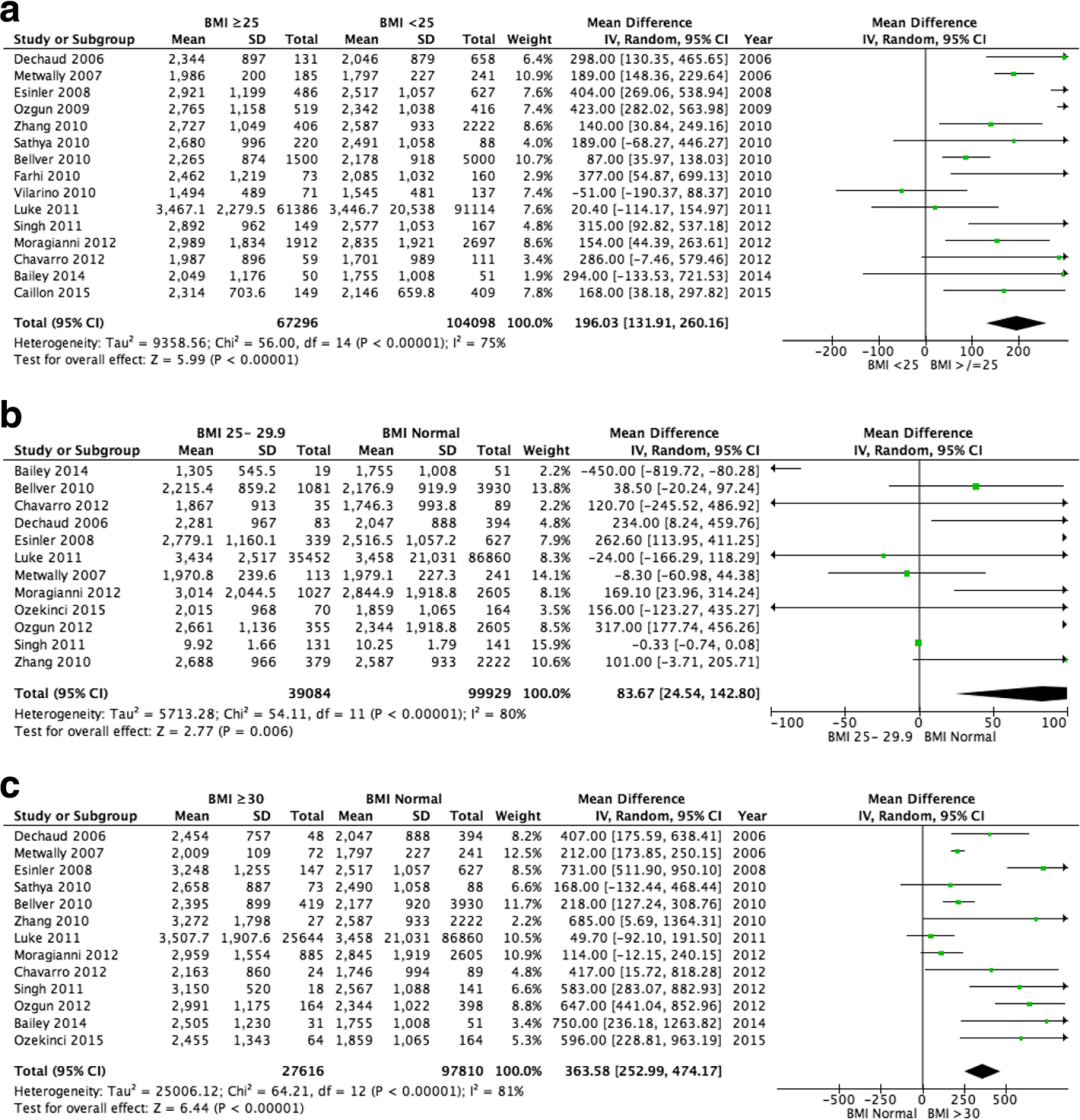
Meta-analysis of total gonadotrophin dose: (**a**) BMI ≥25 kg/m^2^ versus BMI < 25 kg/m^2^; (**b**) Normal BMI versus BMI 25–29.9 kg/m^2^; (**c**) Normal BMI versus BMI ≥30 kg/m^2^

Women with a BMI ≥25 kg/m^2^ required significantly larger total gonadotrophin dosages than women with a BMI < 25 kg/m^2^ (15 studies pooled, weighted mean difference [WMD] 196.03iu, 95% CI 131.91–260.16, *p* < 0.00001; Fig. 5a). However, significant heterogeneity (I^2^ = 75%, *p* < 0.00001) was present between the studies.

Women with a BMI 25–29.9 kg/m^2^ were demonstrated to require significantly higher total gonadotrophin dosages than women with a normal BMI (12 studies pooled, WMD 83.67iu, 95% CI 24.54–142.80, *p* = 0.006; Fig. 5b). However, significant heterogeneity (I^2^ = 80%, *p* < 0.00001) existed between the studies.

Furthermore, increased total dosages of gonadotrophin was documented for women with a BMI ≥30 kg/m^2^ when compared to women whose BMI fell into the normal category (13 studies pooled, WMD 363.58iu, 95% CI 252.99–474.17, p < 0.00001; Fig. 5c). However, significant heterogeneity (I^2^ = 81%, *p* < 0.00001) was present between the studies.

#### Duration of gonadotrophin stimulation

**Fig. 6:**
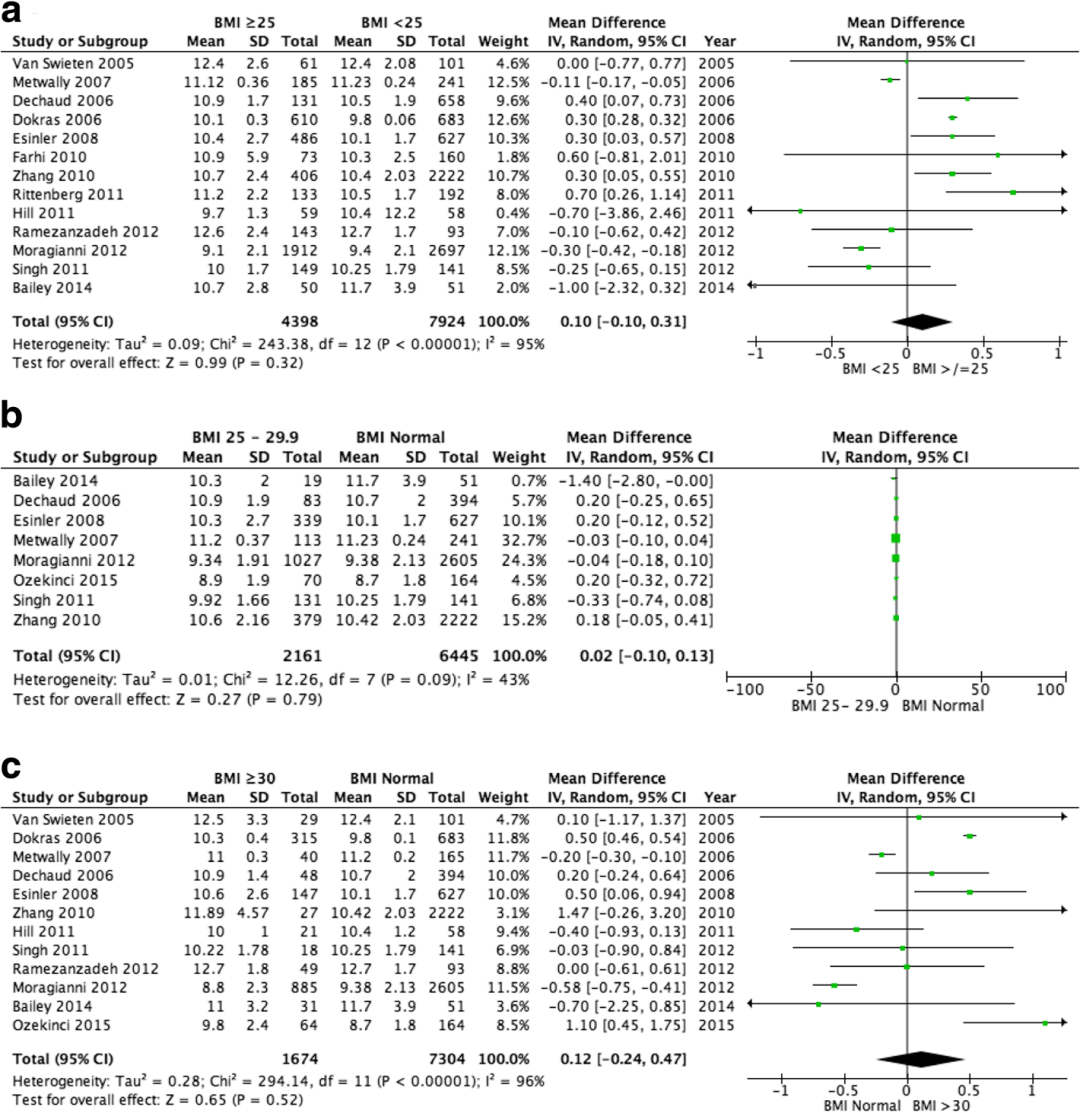
Meta-analysis of duration of gonadotrophin stimulation: (**a**) BMI ≥25 kg/m^2^ versus BMI < 25 kg/m^2^; (**b**) Normal BMI versus BMI 25–29.9 kg/m^2^; (**c**) Normal BMI versus BMI ≥30 kg/m^2^

No significant difference in duration of stimulation therapy was documented between women with a BMI < 25 kg/m^2^ or ≥25 kg/m^2^ (13 studies pooled, WMD 0.10, 95% CI -0.10-0.31, *p* = 0.32; Fig. 6a), however significant heterogeneity (I^2^ = 95%, *p* < 0.00001) existed between the included studies.

Furthermore, no significant difference was seen for duration of gonadotrophin stimulation between women with a BMI 25–29.9 kg/m^2^ versus a normal BMI (8 pooled studies, WMD 0.02, 95% CI -0.10-0.13, *p* = 0.79, I^2^ = 48%; Fig. 6b) or for women with a BMI ≥30 kg/m^2^ versus a normal BMI (12 pooled studies, WMD 0.12 95% CI -0.24-0.47, *p* = 0.52; Fig. 6c), however significant heterogeneity (I^2^ = 96%, *p* < 0.00001) was noted between the studies for the latter comparison.

## Discussion

Public funding for fertility services within the United Kingdom is limited, and therefore, strict guidance exists regarding who can be offered treatment under the National Health Service (NHS). Body mass index (BMI) is a universal criteria adopted by both the public and private sector. This study addresses an important aspect of the impact of a raised BMI on fertility treatment outcomes.

We standardise the analysis of the data by only including studies incorporating the WHO BMI criteria; the current reference point for clinicians and clinical commissioning groups in ascertaining which group of patients should receive treatment. This study is an update of the previous systematic review performed in 2010, with the inclusion of a larger number of cycles from central databases such as the Society for Assisted Reproductive Technology (SART).

This systematic review and meta-analysis has clearly highlighted the negative impact of a raised BMI on the outcomes following ART treatment, with documented lower success rates and higher rates of miscarriages as well as higher total dosage of gonadotrophin usage with no effect on the duration of stimulation. The latter may have been balanced by higher dosages of treatment which can also have a cost implication. However, as most studies have included a BMI category of < 25 kg/m^2^, which would also include underweight women with a BMI < 18 kg/m^2^, the detrimental effects of which have been addressed in a number of previous studies, a risk of bias cannot be confidently excluded. This has been addressed through the inclusion of studies allowing for a sub-group analysis of women with a normal BMI with overweight and obese women.

The presented data is able to demonstrate statistical significance with low clinical heterogeneity for a number of factors reflective of success through ART treatment. Despite this, caution is advised for interpretation of the presented information as only a few of the included studies controlled for confounding factors such as age, smoking and duration of infertility. In order to reduce further clinical heterogeneity, studies not incorporating the WHO classification for BMI and paternal BMI were excluded.

The included studies were considered relevant if they conformed to the WHO classification of BMI, despite this, a considerable amount of methodological and clinical heterogeneity existed. The level of statistical heterogeneity for the primary outcome measure live birth rate and secondary outcome measures clinical pregnancy rate and miscarriage rate were limited. However, despite a significant increase in total gonadotrophin dosage requirements with increasing BMI categories, the studies demonstrated significant statistical heterogeneity, limiting their value.

The presented data can act as an aid in the counselling of subjects secondary to a clear impact on ART outcomes being demonstrated across all BMI categories. The evidence supports the government’s stringent allocation of funding when resources are significantly limited.

A raised BMI impacts reproductive health at the pre and post embryological stage of development, affecting oocyte quality and the endometrial environment [2].

A recent meta-analysis and systematic review by Best et al., [5] has demonstrated that weight loss can improve pregnancy rate and ovulatory status with a trend favouring spontaneous conception. However, these effects have not been seen through ART. Of note, miscarriage rates were unaltered with a change in weight.

Besides the reproductive health effects of a raised BMI, clinicians should also be aware of the increased rate of pregnancy complications such as pregnancy induced hypertension, pre-eclampsia and gestational diabetes in women with a raised BMI. Women are also at an increased risk of an emergency caesarean section with increasing BMI [6, 7].

A holistic approach should be used when counselling patients seeking ART treatments using an open discussion method to inform patients of the effects of raised BMI on ART and obstetric care. This will allow couples to make an informed decision and to take ownership of their well-being.

## Conclusion

This systematic review and meta-analysis further emphasises the negative impact of a raised BMI on ART outcomes. However, the underlying pathophysiology is beyond the scope of this systematic review and will need to be evaluated in future studies. The quality of this systematic review would be further improved if future study designs included the WHO classification of BMI and controlled for confounding variables.

## Abbreviations

ART: Assisted reproductive technology
BMI: Body Mass Index
FSH: Follicle stimulation hormone
GIFT: Gamete intra-Fallopian transfer
HCG: Human chorionic gonadotrophin
ICSI: Intracytoplasmic sperm injection
IVF: In vitro fertilization
MeSH: Medical subject headings
NHS: National Health Service
OHSS: Ovarian hyperstimulation syndrome
SART: Society for Assisted Reproductive Technology
